# Violence against children and natural disasters: A systematic review and meta-analysis of quantitative evidence

**DOI:** 10.1371/journal.pone.0217719

**Published:** 2019-05-30

**Authors:** Ilan Cerna-Turoff, Hanna-Tina Fischer, Susannah Mayhew, Karen Devries

**Affiliations:** 1 Department of Global Health and Development, London School of Hygiene and Tropical Medicine, London, United Kingdom; 2 Department of Population and Family Health, Mailman School of Public Health, Columbia University, New York City, New York, United States of America; Washington University in St. Louis, UNITED STATES

## Abstract

**Objectives:**

Reviews of violence against children in disaster settings focus on armed conflict. Little is understood about natural disasters which has implications in planning humanitarian response. We examined the magnitude and direction of the association between exposure to natural disasters and physical, emotional, and sexual violence against children, and assessed the quality of the evidence.

**Methods:**

We searched 15 health and social science databases from first record until May 16, 2018. Publications describing all types of quantitative study design were eligible for inclusion. We presented study characteristics and quality in a narrative form and generated pooled estimates using a three-level random effects model. We evaluated Cochrane’s *Q* with *p*-values below 0.10 and radial plots to assess heterogeneity. Planned subgroup analyses explored differential results by violence form, study design, and analysis method.

**Results:**

11 publications met inclusion criteria. The majority were cross-sectional studies examining physical or sexual violence in the United States. We found no evidence of a consistent association or directional influence between natural disasters and violence against children. Combined categorical violence outcomes had substantial heterogeneity [*Q* (df = 66) = 252.83, *p* < 0.001]. Subgroups without evidence of heterogeneity had confidence intervals that included a possible null effect. Our findings were mainly limited by inconsistencies in operational definitions of violence, a lack of representative sampling, and unclear establishment of temporal order between natural disaster exposure and violence outcomes.

**Conclusions:**

Based on the available evidence, we cannot confidently conclude that natural disasters increase the level or severity of violence against children above non-disaster settings, however heterogeneity and study quality hamper our ability to draw firm conclusions. More nuanced and rigorous research is needed to inform practice and policy as natural disasters increasingly affect human populations.

## Introduction

Natural disasters are increasing in severity and frequency due to climatic changes [1–3] and adversely affecting a greater number of people worldwide [4,5]. In 2016, forced displacement from natural disasters accounted for 24.2 million new displacements across 118 countries and territories; three times the number of people displaced from conflict-related events in the same period [6]. The United Nations International Children Fund (UNICEF) estimates that approximately 535 million children were affected by natural disasters and other forms of disasters in the same year [7].

A widely assumed negative health outcome of natural disasters is violence against children. Natural disasters elevate known risk factors for violence in non-disaster settings; such as, caregiver stress, food insecurity and poverty, community substance abuse, and mental health disorders in both caregivers and children [8–10]. These risk factors do not occur in isolation, and their cooccurrence can lead to further forms of violence [11]; parental maltreatment in childhood, for instance, is associated with experiences of dating violence in adolescence [12].

Disaster settings, whether instigated by a natural disaster or armed conflict, additionally contain unique characteristics that may lead to increased violence. During disasters, the collapse of systems of social control and policing, mass displacement, and the separation of children from caregivers can heighten the risk of sexual violence and criminality [13–15]. The social support deterioration model posits that trauma linked to experiences during the disaster event or its aftermath may disrupt family functioning and erode existent social support structures, leading to increased violence within children’s established care networks [16]. Domestic or intimate partner violence within households likewise can be exacerbated or produced by the breakdown of family function [17–23]. After Hurricane Katrina, Schumacher [24] found that psychological victimization significantly increased by over ten percent for both men and women and physical violence against women doubled. Violence against women and violence against children tend to co-occur in households, and even when indirect, witnessing domestic or intimate partner violence negatively impacts children’s mental health and increases their probability of perpetrating physical and sexual violence in the future [25,26].

While natural disasters and armed conflict share many underlying drivers, they may differ in how they affect violence against children. In a literature review and statistical analysis of disaster victims, Norris [27] found that survivors of armed conflict and terrorism had worse psychological outcomes than those who survived natural disasters when he pooled 160 individual samples from 29 countries. Norris’ findings may imply that human response to natural disasters is distinct from that of armed conflict. Given the intersection between poor mental health and violence against children [9,28], the quantity of violence that is attributable to individual psychopathology on average would likely be higher in situations of armed conflicts than natural disasters.

Unlike violence initiated by humans, natural disasters may leave community trust intact or bolster cooperation. In some settings, natural disasters are associated with reductions in violent crime [29–31], increases in family functioning [32,33], and prosocial behavior in communities and families [34–36]. One such study on communities affected by Hurricane Andrew found that one-third of respondents reported less stress with their neighbors than before the disaster, and 90% felt that the sense of sharing had been high in the neighborhood immediately after the hurricane and one year later [37]. Multiple disaster researchers over the years have hypothesized that the pattern of altruism and community cohesion after a disaster is shaped by pre-disaster social organization [38–41]. However, armed conflict is the result of underlying social inequality and tension [42–44] and occurs within a cycle of mass violence—44% of states that experienced civil war relapse into combat within five years [45]. The starting point for armed conflict typically is a weakened social structure which may be less conducive to protecting children from violence.

Religiosity and a positive interpretation of the disaster event may similarly play a role in moderating the effect of natural disasters on violence against children. A body of research has illustrated that religiosity increases following natural disasters. A recent study of five waves of data from the World Values Study and European Values Survey in 96 countries overlaid with spatial data from natural disasters indicated that so-called “religious coping”, or increased religiosity as a means of bearing an unpredictable and unbearable situation, was highly associated with earthquakes [46]. The effect of being spared from perceived “acts of God” may uniquely lead to better individual mental health and greater unity of families and communities as compared to situations of armed conflict. While a distinct pathway, the underlying mechanism is complicated in that religious coping seems to improve psychological outcomes inconsistently and solely among those with a positive outlook on their situation. In contrast, feeling “punished by a divine force” increases posttraumatic stress disorder, depressive symptoms and other psychopathology [47,48]. The difference in mental health outcomes when individuals have a positive or negative interpretation is apparent in Muslims affected by the 2005 Pakistan earthquake [49], Christians exposed to Hurricane Katrina and Rita in the United States [50,51], and Buddhists after the 2004 Indian Ocean tsunami in Sri Lanka [52]. The crux of how natural disasters may differ from armed conflict and lead to differences in violence patterns may be that individuals regardless of religious belief or affiliation can possess positive beliefs that natural disasters have greater meaning which transcends human control and action.

Despite the implications for public health, a limited body of research exists on the relationship between natural disasters and violence against children. This paper fills in a gap in the literature as the first known systematic review and meta-analysis to examine the magnitude and direction of association between natural disasters and violence against children. The review aims to analyze the existing quantitative evidence to understand how associations differed across each form of violence and assess the quality of sampling and study design, measurement, and statistical analysis.

## Methods

### Systematic literature review

We conducted a systematic review to identify studies that provided an estimate of the magnitude and direction of association between natural disasters and violence against children. Children were defined as individuals under the age of 18, and the definition of violence encompassed physical, emotional, and sexual violence as well as bullying, maltreatment, and interpersonal violence. Witnessing domestic or intimate partner violence were categorized as a form of emotional violence in this review, given the indirect but harmful effect on children and its inclusion within family violence literature as a form of violence against children [26,53,54]. The specific definition of violence was extracted for each article, and we operationalized general violence terms by applying the UNICEF Hidden in Plain Sight report’s [55] definitions (Table 1). The United Nations International Strategy for Disaster Reduction (UNISDR) [56] broadly defines a disaster as: “A serious disruption of the functioning of a community or society involving widespread human, material, economic or environmental losses and impacts, which exceeds the ability of the affected community or society to cope using its own resources” (p. 9). In this review, the term “natural disaster” encompasses disasters triggered by hydrometeorological, geophysical, and climatological events [57]. The category purposefully includes both slow and rapid onset disasters but excludes manmade disasters, such as nuclear failures or oil spills. We constructed the violence and children Boolean search operators by adapting terms from several other reviews [58–61]. The search strategy for Medline/PubMed is provided for reference (S3 Table).

**Table 1:** Operational definitions of violence.

Physical violence	“…all corporal punishment and all other forms of torture, cruel, inhuman or degrading treatment or punishment as well as physical bullying and hazing by adults or by other children”
Emotional violence	“Psychological maltreatment, mental abuse, verbal abuse and emotional abuse”
Sexual violence	“…any sexual activities imposed by an adult on a child against which the child is entitled to protection under criminal law” or “…committed against a child by another child if the offender is significantly older than the victim or uses power, threat or other means of pressure”

Two authors (ICT and HTF) searched 15 academic databases for articles published between the earliest logged record and May 16, 2018 (S8 Table). We restricted articles to the English language for all geographical locations. Rayyan software for systematic reviews was used to manage returned citations, remove duplicates, and blind each author’s initial screening decisions on article inclusion [62]. Each author independently screened titles and abstracts against standardized inclusion and exclusion criteria (S9 Table). Once unblinded, ICT and HTF reconciled any conflicting decisions for inclusion or exclusion (17 articles in total). The full articles were retrieved if they referenced natural disasters and violence against children in the title or abstract after reconciling any conflicting decisions with a third author (KD), if needed. We extracted key information from articles in duplicate using pilot tested forms and jointly reconciled decisions. Although ICT and HTF were not blinded to the names of the publication authors or their institutional affiliation during data extraction, this information was not examined in any way to make decisions on final article inclusion or to assess quality. ICT contacted first and last authors of included publications when they were missing key information on study design and outcome measures. One of the contacted authors provided additional bivariate estimates of the effect of the Indian Ocean tsunami on violence against children which were absent in the original publication [63].

We independently assessed study quality by using the National Institute of Health Quality Assessment Tool for Cohort and Cross-sectional and Case-Control Study Designs [64] to critically compare the risk of bias in the included studies (S5–S6 Tables). Following the Cochrane Handbook for Systematic Reviews of Interventions [65], we employed these checklists to aid in comparing study quality rather than as tools for inclusion and exclusion decisions. We furthermore reviewed quality dimensions that were important to our research question. These factors include temporality; variability in operational definitions and measurement; reporting biases and missing data; representativeness; and statistical adjustments and exclusion of moderators. ICT and HTF reviewed the score for each question within assessment tools together when quality scores differed and reconciled differences in discussion—all studies were rated differently by at least one question of the assessment tools. The review process abided by the Cochrane Handbook for development and execution of the search process [65] and the Preferred Reporting Items for Systematic Reviews and Meta-Analyses (PRISMA) guidelines for reporting [66]. The review protocol was prospectively registered in PROSPERO, number: CRD42018087862, and is attached (S2 Table).

### Data synthesis

We synthesized the data in a narrative form followed by generating pooled estimates of violence against children following natural disasters. Salient study characteristics were compared and contrasted, including geographic location, population of children, study design, measurement, and statistical adjustment and interaction. We applied three-level random effects models to generate pooled odds ratios (OR) for categorical violence outcomes, with 95% confidence intervals (CI). Continuous violence outcomes were not statistically synthesized due to the small sample size and limited number of studies identified. This decision builds upon recent guidance that random effects meta-analysis of continuous measures should have at least five estimates to achieve consistent statistical power [67]. A three-level random effects model or multi-level meta-analysis produces generalizable results from a limited number of studies, which was particularly warranted for this review. The model allowed for selection of more than one estimate per study, reducing second order sampling bias and increasing power [68], and the random effects reduce possible errors caused by overweighting studies with large sample sizes or by making assumptions of a common underlying population [69]. We assessed heterogeneity using Cochrane’s *Q* statistic and employed a *p*-value of 0.10 to determine statistically significant heterogeneity beyond sampling bias [65]. Visual inspection of radial plots accompanied heterogeneity testing, because even when non-significant, the *Q* statistic has low power to detect effects. When evidence of low heterogeneity, we visually examined contour-enhanced funnel plots to determine the possible presence of publication bias.[70] Analysis was conducted using the metafor package in R v.3.3.3 [71,72].

The statistical analysis followed a set protocol, using assessments of heterogeneity to determine subsequent steps in analyses and reporting. ICT initially analyzed the pooled estimate of all forms of categorical and continuous violence respectively and proceeded to subgroup analysis of the categorical violence outcomes by violence type, unadjusted and adjusted outcome measures, studies without lifetime experiences of violence, and studies that compared pre- and post-measures of violence. Within the subgroup analysis of violence types, ICT examined the overarching categories of physical, emotional, and sexual violence as well as typologies of violence within each category, such as dating violence, when comparable across multiple studies. Emotional violence was analyzed with and without the inclusion of witnessing domestic or intimate partner violence to explore any possible differences. Continuous outcomes could not be stratified for further analysis, given the scarcity of estimates. When subgroups did not indicate excessive heterogeneity and had adequate sample size to produce robust results, ICT generated pooled estimates and examined the presence of possible publication bias in contour-enhanced funnel plots.

## Results

### Study characteristics and quality

**Fig 1:**
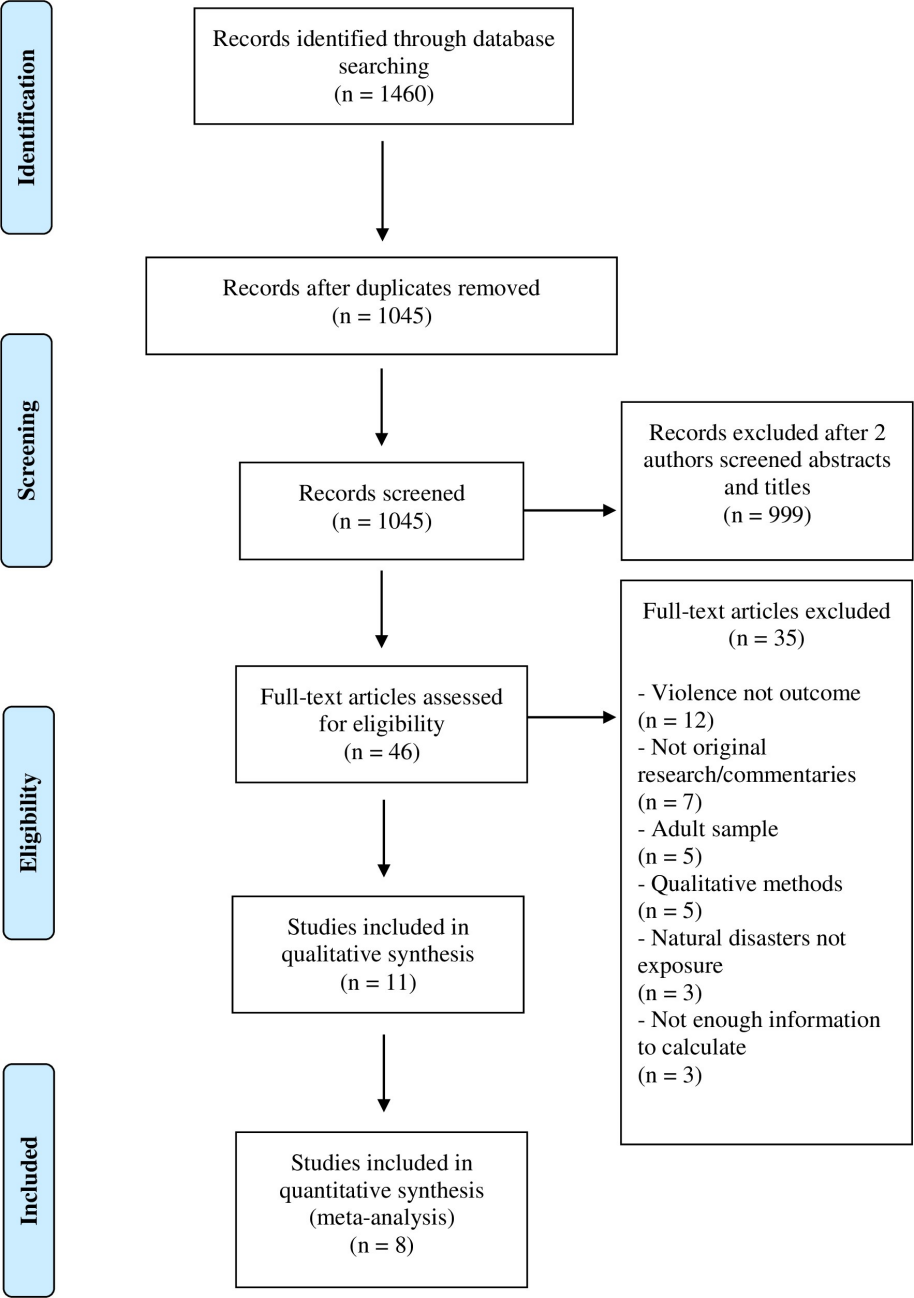
Flowchart of included quantitative studies.

A total of 11 articles met the inclusion criteria for data extraction (Fig 1). Seven of the included studies were from the United States [73–79], two were from Sri Lanka [63,80], one was from Bangladesh [81], and one was from Haiti [82]. Concurrent or recent armed conflict was solely present in Sri Lanka [83], and Haiti had experienced recent political violence [84]. The majority of studies sampled school-going children in addition to their caregivers [63,76–80], and the age of children spanned from 0 to 18 years of age [63,73–82].

Six of the included studies were cross-sectional [63,73,78,80–82], and one study used a case-control design [76]. The remaining studies applied designs that can aid in isolating changes ascribable to natural disasters on the population level—interrupted time series design [74], pre-post quasi-experimental design with a control group [75,79], and repeat cross-sectional design [77]. Participation rates in the studies ranged considerably, from 36% [76] to 100% [63]. A lack of representativeness in sample selection decreased rigor in some studies. One of the cross-sectional studies used purposeful sampling of respondents [82], and two studies sampled controls from populations that differed in ways that may have affected the outcome and at a different timeframe from the cases [76,79].

### Disaster measures

Several different disaster events were described, of which 50% were hurricanes [63,73–82]. All types of disaster events had a rapid onset, with the exception of one study that included any form of disaster exposure [73]. Exposure to disasters was often measured ecologically as being within a certain area at the time of disaster [74,75,77,79,81] or in self-report using scales [63,73,76,80]. Two articles used proxy measures of living in camps and evacuation status to measure disaster exposure [78,82]. In the case of Bangladesh, Biswas [81] combined two separate flood events that occurred in succession over a short timeframe for the categorization of exposure, and Curtis [74] examined three disasters in the United States—Hurricane Hugo, the Loma Prieta earthquake, and Hurricane Andrew—at multiple time points.

### Violence measures

The most common type of violence outcome described in the articles was physical violence and corporal punishment [63,73–76,82], followed by sexual violence [63,73,74,82]. Several studies relied on administrative data or reports from caregivers which tend to underestimate violence in stable settings [85,86]. The studies varied in their practice of recording perpetrator types—some studies did not explicitly document perpetrators for all or some violence measures [73–75,77] while others employed detailed categorization [63,81]. The most commonly documented perpetrator types was parents [63,76,81,82] or intimate partners [77,78,82]. Four studies specified a timeframe in their violence measurement that followed natural disaster exposure [74–76,81]. In all other cases, the authors either measured violence as lifetime experiences, did not specify when violence occurred, or sampled at a timepoint when a proportion of the violent acts could have occurred before the disaster [63,73,77–80,82]. Violence outcomes were measured anywhere from weeks [81] to years [77] afterwards. In all but three studies, the authors used binary measures in quantifying violence as an outcome [73–75,77–79,81,82].

### Statistical adjustment and interaction

Most studies statistically adjusted for some important confounders that are typical in violence studies and public health, such as gender, race/ethnicity, and the age of the child. However, it is important to note that two authors adjusted for factors that could be on the causal pathway between natural disaster exposure and experiences of violence in childhood. Biswas [81] included violence against mothers during disasters, and Catani [80] incorporated a variable on if the father or mother was deceased without specifying if the death occurred during or after the disaster. No article included moderating variables, such as social support and family functioning, community cohesion, and religious coping style and ascribed meaning [63,73–82] (Table 2). A full description of study characteristics is found in the supplemental materials (S4 Table).

**Table 2:** Quality characteristics of included studies.

Data source	Disaster measured before violence (y/n)	Disaster measured ecological (y/n)	Period of time between disaster and violence	Reporting	Participation rate (%)	Random sample (y/n)	Adjusted for confounders	Adjusted for factors on the casual pathway
Becker-Blease [73]	N	N	Any period during lifetime	Both	70.3%	Y	Y	N
Biswas [81]	Y	Y	1 week-1 month	Mother	65.0%	Y	Y	Y
Catani [80]	N	N	Any period during lifetime	Self	N/R	Y	Y	Y
Curtis [74]	Y	Y	3, 6, and 11 months	Administrative review	N/A	N/A	Y	N
Keenan [75]	Y	Y	~ 0–6 months and subsequent 6–21.5 months	Administrative review	N/A	N/A	Y	N
Kelley [76]	Y	N	3–7 months	Mother	36.0%	N/A	N	N/A
Madkour [77]	Both	Y	Any period during lifetime (sexual); ~ 8–20 months (physical)	Self	61.0%	Y	Y	N
Sloand [82]	N/R	N/R	12–36 months	Self	N/R	N	N	N
Sriskandarajah [63]	N	N	Any period during lifetime	Self, mother, and father	100.0%	Y	N	N
Temple [78]	N	N	Timeframe overlaps with 6 months pre and 6 months post	Self	76.0%	N/A	Y	N
Terranova [79]	N	Y	Unspecified timeframe	Self	60.0% (time 1); 62.0% (time 2)	N/A	Y	N

### Statistical synthesis

#### Categorical violence outcomes

**Fig 2:**
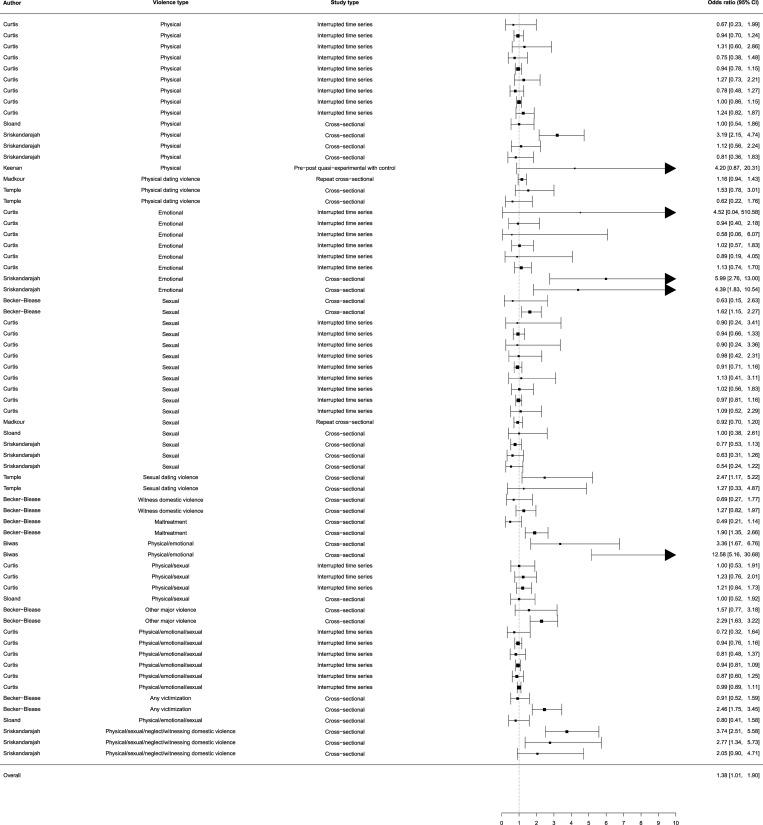
Forest plot of categorical violence outcomes. A three-level random effects model of the association between natural disasters and any form of violence against children. Multiple estimates were drawn from the same study if the author’s name is used in more than one row. Curtis [74] analyzes three separate disasters within their article. All estimates were converted to OR for categorical violence outcomes, with 95% CIs.

The eight studies with categorical violence outcomes included 67 estimates and had a total sample size of 332,882 individuals. Natural disasters slightly increased the overall odds of violence (pooled OR 1.38, 95% CI 1.01–1.90) (Fig 2). Statistical testing however identified substantial heterogeneity (*Q* (df = 66) = 252.83, *p* < 0.001). The presence of considerable heterogeneity was confirmed in a visual inspection of radial plots (S1 Fig).

#### Subgroup analysis

Four of the 18 subgroup analyses without evidence of excessive heterogeneity and sufficient sample size had confidence intervals that crossed 1.00 which indicates no clear directional change or evidence of effect (Table 3). Visual inspection of funnel plots did not indicate the presence of publication bias in any subgroup. The forest, radial, and funnel plots for each subgroup are included in the supplemental materials (S2–S13 Figs).

**Table 3:** Subgroup analysis by study design and violence outcome.

Subgroup	Number of estimates	Sample size	Pooled OR (95% CI)	95% CI	Cochrane’s *Q*	*p*-value
Excluded lifetime–physical violence	14	93,878	1.05	0.91–1.20	12.307 (df = 13)	*p* = 0.503
Pre-post– all violence outcomes	36	271,001	1.01	0.90–1.15	16.754 (df = 35)	*p* = 0.996
Pre-post–physical violence	11	92,752	1.05	0.89–1.24	10.033 (df = 10)	*p* = 0.438
Pre-post–sexual violence	10	59,867	0.95	0.85–1.06	0.524 (df = 9)	*p* = 1.000

All subgroup analyses are for categorical violence outcomes

## Discussion

We found no evidence of a consistent statistical association and directional influence between natural disasters and violence against children. The study characteristics and quality however tempered our conclusions. Most studies were conducted in the United States where established infrastructure and access to services may result in inherently lower violence estimates than disaster-prone areas of the world with fewer resources [73–79]. The studies tended to treat children as one group, without stratifying or statistically adjusting for subpopulations, which possibly masked elevated violence among certain groups of highly vulnerable children [87–89]. In particular, sampling children in schools likely resulted in violence estimates that were lower than those for out-of-school children who may be more vulnerable to violence [90,91]. Low-income families that are evacuated are less likely to return to disaster-affected areas [92], leading to samples that potentially missed a group of children who may have higher levels of violence [89]. Cross-sectional surveys additionally cannot identify changes from pre-disaster estimates of violence, and low participation rates and non-representative sampling methods frequently lead to biases.

This review adds to the body of knowledge on violence against children in disaster settings. No other review has expressly examined natural disasters and violence against children [21,93,94]. In a systematic review on violence against children [93] and another systematic review of gender-based violence [21], both reviews were almost entirely composed of violence studies in situations of armed conflict. This review distinguishes natural disasters from armed conflict to explore differences in quantitative effects by violence type, measurement, and study design. Past reviews have expressed that violence trends were impossible to determine but were likely high on the population level [21,93]. Our findings may indicate that natural disasters have a unique pattern of violence from that of armed conflict which merits further study.

Our ability to draw firm conclusions was limited by the measurement and analysis methods of the included studies. The most common metric for measuring natural disaster exposure was geographic proximity which, depending on disaster type and severity, can be a weak proxy for direct exposure since not all people within a physical area are necessarily exposed [74,75,77,79,81]. Few studies used exactly comparable violence definitions. The reliance on caregiver reports and administrative data likely would have underestimated the measurement of violence in many studies [74–76,81], and past research has shown that child sexual violence rates may be greater than 30 times higher [85], and physical abuse rates may be more than 75 times higher in self-reporting [86]. The measurement of violence was restricted to acts committed by a limited range of actors and most commonly parents [63,76–79,81]. While the restriction often matched the goals of the individual study, the results would likely underestimate the total amount of violence that children experienced. Meaningful associations may have been diluted by the lack of establishment of a clear temporal order between natural disasters and violence and by the measurement of violence multiple years after the disaster in some studies. The reliance on binary variables for measuring violence outcomes possibly missed subtle changes in modeling the effect of natural disaster exposure. Finally, in some studies, the statistical adjustment for variables on the causal pathway could have decreased the estimated association between natural disasters and violence, and the exclusion of moderating factors could have led to inaccuracies in measurement.

The pooled estimate of combined violence outcomes indicated substantial heterogeneity, and subgroup analysis did not provide a clear explanation of the source of the variability. The majority of the heterogeneity was likely due to the inconsistencies in the methodologies of the included studies. The four subgroups that were fully analyzed similarly indicated no clear change in the direction of the association between natural disasters and violence against children. Publication bias was unlikely in the subgroup analyses. The funnel plots exhibited an absence of studies with low precision—small sample sizes and large variance—but they were symmetrical and did not have patterns of missingness for negative or positive results. This finding implies that factors other than publication bias, such as variability in study quality, are more likely present [95].

### Strengths and limitations

This review searched global databases to produce the first systematic review and meta-analysis on the relationship between natural disasters and violence against children. Despite the comprehensiveness and statistical rigor of this review, it has several limitations. The main limitation is that our search concentrated on health and social science databases. The relationship between violence against children and natural disasters cross-cuts multiple disciplines, and relevant articles may have appeared in criminology, social work, or disaster response databases beyond the scope of our search strategy. We however attempted to search a multitude of prominent databases where articles on violence would likely be referenced. Additionally, it is possible that we missed relevant publications due to the variable terminology used to describe violence, populations of children, or natural disasters. A further limitation is that we did not include non-English sources. We therefore potentially did not identify relevant studies reported in non-English settings where natural disasters frequently occur.

### Implications

Few studies have measured violence against children following natural disasters, and this review is the first systematic attempt to quantify and understand the underlying relationship. At various junctures, the World Health Organization (WHO) and academic literature have called for more evidence on this topic [74,96,97]. Despite the appeals, quantitative studies on violence against children in the aftermath of both natural disasters and armed conflict remain scarce and continue to suffer from quality issues [21,93]. At minimum, more high-quality research is needed to understand how natural disasters are associated with violence against children and to distinguish how the relationship may differ from situations of armed conflict.

Our findings challenge assumptions that violence will be escalated above normal levels following a natural disaster. Violence against children is high globally regardless of the occurrence of a disaster, and services for children are always critical in all settings [98,99]. However, we cannot confidently conclude that natural disasters increase the level and severity of violence against children above that found in non-disaster settings, based on the scope and quality of the available evidence. We do not clearly understand which types of violence are most likely to increase, for which groups of children, and in what contexts following natural disasters. These differences may necessitate specialized services to certain children at specific times. We may need to tailor interventions differently than our current practices to maximize our effort.

We are entering a time when global climate change is spurring increased and more severe natural disasters [1–3] and affecting a greater number of people globally [4,5]. In particular, lower- and middle-income countries are disproportionately affected by natural disasters—they represent 11% of the population exposed to natural disasters but 53% of the casualties [100]. Future natural disasters may therefore occur in places where health and protection systems are less developed [101,102], and the sudden influx of people needing services and increased barriers to service provision are likely to further burden overstretched healthcare providers and social workers [103,104]. It is imperative that we understand the fundamental relationship between natural disasters and violence against children to create effective policies and to allocate limited resources in health and child protection systems based on evidence.

We identified several areas for recommended future research in this review. More research is needed about lower- and middle-income countries that face disproportionate vulnerabilities to natural disasters [100]. Contextual understanding of the effect of past or concurrent armed conflict, political violence, and seasonal and successive disaster events on violence against children would aid in isolating and differentiating natural disasters from other environmental disruptions. In addition, all of the natural disasters in this review had a rapid onset. Investment in better understanding slow-onset disasters is merited. Future research should explore factors that are protective and moderate violence in natural disaster contexts, as these factors may be distinct from those found in man-made disasters. We have reason to consider social support and family functioning, community cohesion, and religious coping style and ascribed meaning as starting points [32,37,51,105,106].

## Conclusions

More high-quality and nuanced research is needed on the association between natural disasters and violence. Without scientifically examining the relationship in a rigorous manner, we negate the possibility of understanding the effect of natural disasters on violence against children and in identifying populations that are most at risk for specific forms of violence. In a limited funding environment with multiple priorities, targeting effective interventions to the most vulnerable populations is essential. As natural disasters increasingly affect human populations, we must better understand the underlying relationship with violence to protect children and improve human health.

## Supporting information

S1 Table PRISMA checklist. (DOCX)

S2 Table Review protocol. (DOCX)

S3 Table Medline search strategy. (DOCX)

S4 Table Descriptive analysis of included studies. (DOCX)

S5 Table Risk of bias results for cross-sectional and cohort studies. (DOCX)

S6 Table Risk of bias results for case-control studies. (DOCX)

S7 Table Data used in R for meta-analysis. (XLSX)

S8 Table Data repositories searched. (DOCX)

S9 Table Inclusion and exclusion criteria. (DOCX)

S1 Fig All categorical violence outcomes radial plot. (EPS)

S2 Fig Physical violence without lifetime measures forest plot. (EPS)

S3 Fig All violence outcomes with pre-post design forest plot. (EPS)

S4 Fig Physical violence with pre-post design forest plot. (EPS)

S5 Fig Sexual violence with pre-post design forest plot. (EPS)

S6 Fig Physical violence without lifetime measures radial plot. (EPS)

S7 Fig All violence outcomes with pre-post design radial plot. (EPS)

S8 Fig Physical violence with pre-post design radial plot. (EPS)

S9 Fig Sexual violence with pre-post design radial plot. (EPS)

S10 Fig Physical violence without lifetime measures funnel plot. (EPS)

S11 Fig All violence outcomes with pre-post design funnel plot. (EPS)

S12 Fig Physical violence with pre-post design funnel plot. (EPS)

S13 Fig Sexual violence with pre-post design funnel plot. (EPS)
