# Supplementary material for: Violence against children and natural disasters: A systematic review and meta-analysis of quantitative evidence
Source: PLoS One. 2019 May 30;14(5):e0217719. doi: 10.1371/journal.pone.0217719 (PMC6542532; doi:10.1371/journal.pone.0217719)
Supplement: S2 Table — (DOCX) [file pone.0217719.s002.docx]

**S2 Table. Review protocol**

**Title:**

Violence against children and natural disasters: A systematic review and meta-analysis of quantitative evidence

**Language:** English

**Publication period:** database first publication date to May 16, 2018 (present)

**Review Questions/Objectives:**

- To identify the magnitude and direction of the association for physical, emotional and sexual violence against children after natural disasters
  - What is the magnitude and direction the association for physical, emotional and sexual violence against children after natural disasters?
  - What are the patterns by violence type, population and context?
  - How is exposure to natural disasters measured across studies?
  - What temporal elements of violence post-disaster have been identified?
- To map the evidence and examine the quality of the research
  - What is the current scope and risk of bias for the evidence?

**Sources to be searched:**

The study team will search electronic databases, including: MEDLINE/PUBMED, EMBASE, PsychINFO, International Bibliography of Social Sciences, Global Health, SCOPUS, SciELO, Social Policy and Practice, Web of Science, CINAHL and regional databases (such as, Africa-Wide Information, IndMed, IMEMR, LILACS, MedCarib, WPRIM). The search strategy will include strings related to: 1) children, 2) physical, sexual and emotional violence and 3) natural disasters. The child and violence search strings were adapted from an on-going systematic review of risk and protective factors, which, in turn, was based upon verified search strategies from other reviews. Additional terms for violence and bullying were constructed using two Cochrane review search strategies for similar concepts (Mytton et al 2006; O’Doherty et al 2015). The natural disaster terms were compiled from listing and testing possible natural disaster typologies. The search will be restricted to articles in English and children and adolescents under the age of 18. The searches will run for all articles, from the first record until the present.

**Condition or domain to be studied:** natural disasters, including rapid or slow onset disasters—tsunamis, volcanoes and other weather-related or geological phenomena—and violence against children

**Participants/population:**

Inclusion: children and adolescents under the age of 18

Exclusion: adults (18 years of age and older)

**Intervention(s)/Exposure(s):**

The purpose of the systematic review is to map the evidence and to identify the magnitude and direction of the association and to identify reasons for changes in physical, emotional and sexual violence against children and natural disasters. The systematic review includes both longitudinal and cross-sectional study designs, however, the majority of the evidence is likely to be cross-sectional, given that natural disasters are unpredictable events. Much of the literature has lumped man-made and natural disasters together, with the assumption that they have a common effect of widespread social disruption on all forms of violence.

**Comparator/control:** The exact comparison group, as specified in the article, will be extracted. This could include: a group with no exposure to the natural disaster, pre-disaster measurements for exposed individuals or the underlying population, or no comparison group. If the same underlying population is sampled, the author’s definition of the sampling population will be included in data extraction.

**Types of studies to be included:**

The review will assess quantitative studies of natural disasters with an outcome measure of physical, sexual and/or emotional violence against children, aged 0-17. The included articles will describe original research, which excludes opinion articles and policy reviews. The study team will include only peer reviewed publications.

**Inclusion criteria**

1. Natural disasters are the exposure/treatment
2. Physical, sexual, or emotional violence are an outcome measure of the study
3. Person experiencing violence is a child or adolescents under 18

- Includes both self-reported violence by someone under 18 at the time of interview and violence reported by an adult respondent against someone under 18
- Includes violence against children when it is committed by caregivers who are under 18
- If age range includes people older than 18, include article only if it includes an age breakdown for those under 18

1. Original research in peer-reviewed journal articles

- **Original research** is defined as a new study conducted or a unique analysis of primary or secondary data. It does not include literature reviews

1. All types of quantitative study designs

**Exclusion criteria**

1. Articles that have gang violence, female genital mutilation (FGM) or child labour/exploitation/trafficking/marriage as outcome measures
2. Articles that use neglect as an outcome measure
3. Editorials, policy reviews or general reports that do not introduce new evidence from a specific study
4. Conference abstracts or posters
5. Literature reviews
6. Articles focused on aggression reactions or mental health sequelae in potential perpetrators without mention of an explicit act of violence against a child
7. Articles that did not describe the magnitude or association between natural disasters and violence against children

**Context:** Global

**Primary outcome:**

Physical, emotional and sexual violence are the primary outcomes of interest. Violence can be defined as a wide range of behaviours (i.e. verbal abuse, bullying). We utilized adapted UNICEF broad definitions of physical, emotional and sexual violence to define violence categories for data synthesis and will further extract violence definitions when specified. The definition of each violence category is:

- **Physical violence** is defined as “…all corporal punishment and all other forms of torture, cruel, inhuman or degrading treatment or punishment as well as physical bullying and hazing by adults or by other children” (UNICEF, 2014)
- **Emotional violence** is defined as “psychological maltreatment, mental abuse, verbal abuse and emotional abuse” (UNICEF, 2014)
- **Sexual violence** is defined as “any sexual activities imposed by an adult on a child against which the child is entitled to protection under criminal law” or “…committed against a child by another child if the offender is significantly older than the victim or uses power, threat or other means of pressure” (UNICEF, 2014)

These categories of violence include violence from adults or peers and violence that occurs within and outside of households. It includes violence against married adolescents under the age of 18.

**Secondary outcomes:** None

**Data extraction:**

Titles and/or abstracts retrieved using the search strategy will be screened independently by the primary and secondary author for inclusion. The first round of screening will include articles that: 1) have some form of physical, emotional, sexual or related form of violence as the outcome of interest, 2) focus on children, 17 years and younger, as the recipients of the violence and 3) describe a natural disaster exposure. The two review authors will discuss articles identified in the title and abstract review and if necessary, consult with a third reviewer if discrepancies cannot be resolved.

The full texts of the selected abstracts will then be screened independently. The primary author will create a standardised, electronic data extraction form to synthesize the evidence. The extracted information will include information, such as: pre-disaster setting characteristics, study population, the type of disaster, experience of displacement after disaster, respondent type, violence category (physical, emotional or sexual), definition used for measurement of violence, prevalence/rate/risk/odds (adjusted and unadjusted), and analysis methodology. The data extraction sheet will be pilot tested with a series of relevant articles to ensure that all pertinent information is included. Data will be extracted in duplicate and selected articles will be sorted by overarching and specific study definitions of violence. Any disagreement between the two authors during data extraction will be resolved through discussion with a third reviewer if necessary. Missing data will be solicited from study authors if appropriate.

**Risk of bias (quality) assessment:**

As articles are being selected for inclusion, the two reviewing authors will each separately assess the dimensions for the possible risk of bias. The primary author will adapt questions from a checklist for the assessment of bias in quantitative studies to explore study quality and embed it in the data extraction form. Each aspect for the risk of bias will be categorized as per the recommendations of the Cochrane Handbook for Systematic Reviews of Interventions. Discrepancies in scoring will be discussed between the two reviewing authors. Any possible sources of bias will be checked by the third reviewer.

**Strategy for data synthesis:**

The primary author will provide a separate narrative synthesis of findings, structured around the disaster type, study population, outcome measures and time/location of measurement and analysis methods. Limitations and biases will also be treated in a distinct section. Meta-analysis of the results will also be conducted for the whole dataset and subdivided into analyses by violence type, unadjusted and adjusted outcome measures, studies without lifetime experiences of violence and studies that compared pre- and post- measures. When appropriate, pooled odds ratios for dichotomous outcomes and mean differences for continuous outcomes with confidence intervals will be provided. A final discussion section will discuss possible future areas of development for studying natural disasters and violence against children. It will additionally examine gaps in data collection and the current state of the quantitative evidence.

**Analysis of subgroups or subsets:**

Results from similar subgroups will be compared when possible and if present. The analysis will likewise examine subsets by violence type, statistical analysis methods, and study designs.

**Type and method of review:**

Type

- Epidemiologic
- Methodology
- Systematic Review

Area

- Child health
- Violence and abuse

**Dissemination plans**

We intend to submit this article to a leading journal in the field. We intend to present the results in conferences and disseminate via professional networks of researchers and practitioners.

**Keywords:** Child; child abuse; violence; natural disasters; disasters; emergencies; meta-analysis; systematic review
