# Supplementary material for: Violence against children and natural disasters: A systematic review and meta-analysis of quantitative evidence
Source: PLoS One. 2019 May 30;14(5):e0217719. doi: 10.1371/journal.pone.0217719 (PMC6542532; doi:10.1371/journal.pone.0217719)
Supplement: S3 Table — (DOCX) [file pone.0217719.s003.docx]

**S3 Table**. **Medline search strategy**

| **Search Number** | **Search** | **Results** |
| --- | --- | --- |
|  |  |  |
| **Population String** |  |  |
| #1 | child*.ab,ti. | 1214705 |
| #2 | minor*.ab,ti. | 259764 |
| #3 | schoolchild*.ab,ti. | 12509 |
| #4 | infan*.ab,ti. | 391717 |
| #5 | adolescen*.ab,ti. | 230535 |
| #6 | newborn*.ab,ti. | 147013 |
| #7 | preschool*.ab,ti. | 24135 |
| #8 | pre-school*.ab,ti. | 4399 |
| #9 | kindergarten*.ab,ti. | 5379 |
| #10 | underage.ab,ti. | 949 |
| #11 | youth.ab,ti. | 50522 |
| #12 | youths.ab,ti. | 9935 |
| #13 | baby.ab,ti. | 33627 |
| #14 | babies.ab,ti. | 33149 |
| #15 | prepubescen*.ab,ti. | 912 |
| #16 | pubescen*.ab,ti. | 1982 |
| #17 | schoolage.ab,ti. | 40 |
| #18 | school-age.ab,ti. | 11454 |
| #19 | boy*.ab,ti. | 137310 |
| #20 | girl*.ab,ti. | 131640 |
| #21 | offspring.ab,ti. | 61110 |
| #22 | pediatric*.ab,ti. | 240605 |
| #23 | paediatric*.ab,ti. | 53332 |
| #24 | juvenile*.ab,ti. | 71739 |
| #25 | toddler*.ab,ti. | 8729 |
| #26 | “nursery school*”.ab,ti. | 1024 |
| #27 | “elementary school*”.ab,ti. | 8413 |
| #28 | “high school*”.ab,ti. | 26747 |
| #29 | highschool*.ab,ti. | 40 |
| #30 | “primary school*”.ab,ti. | 10300 |
| #31 | “secondary school*”.ab,ti. | 8660 |
| #32 | daycare.ab,ti. | 1125 |
| #33 | teen.ab,ti. | 4672 |
| #34 | teens.ab,ti. | 5537 |
| #35 | teenage*.ab,ti. | 18827 |
| #36 | “child” [Mesh] | 1934614 |
| #37 | “adolescent” [Mesh] | 1888759 |
| #38 | “infant” [Mesh] | 1111323 |
| **#39** | **or/1-38** | 4091681 |
|  |  |  |
| **Physical/emotional/**  **sexual violence string** |  |  |
| #40 | (physical adj2 violen*).ab,ti. | 2334 |
| #41 | (sexual adj2 violen*).ab,ti. | 3131 |
| #42 | (emotional adj2 violen*).ab,ti. | 271 |
| #43 | (psychological adj2 violen*).ab,ti. | 550 |
| #44 | (mental adj2 violen*).ab,ti. | 472 |
| #45 | (physical adj2 abuse).ab,ti. | 5168 |
| #46 | (sexual adj2 abuse).ab,ti. | 11391 |
| #47 | (emotional adj2 abuse).ab,ti. | 1741 |
| #48 | (mental adj2 abuse).ab,ti. | 1127 |
| #49 | (psychological adj2 abuse).ab,ti. | 786 |
| #50 | (verbal adj2 abuse).ab,ti. | 739 |
| #51 | “child abuse”.ab,ti. | 7805 |
| #52 | (physical adj2 assault*).ab,ti. | 4034 |
| #53 | (sexual adj2 assault*).ab,ti. | 932 |
| #54 | “physical attack*”.ab,ti. | 146 |
| #55 | (severe adj2 punishment).ab,ti. | 82 |
| #56 | (harsh adj2 punishment).ab,ti. | 89 |
| #57 | “corporal punishment”.ab,ti. | 451 |
| #58 | “corporeal punishment”.ab,ti. | 4 |
| #59 | (severe adj2 discipline).ab,ti. | 11 |
| #60 | (harsh adj2 discipline).ab,ti. | 157 |
| #61 | “harsh parenting”.ab,ti. | 233 |
| #62 | harass*ab,ti. | 2918 |
| #63 | incest.ab,ti. | 1276 |
| #64 | maltreat*.ab,ti. | 5885 |
| #65 | “sexual violation*”.ab,ti. | 36 |
| #66 | “forced sex”.ab,ti. | 310 |
| #67 | “coerced sex”.ab,ti. | 38 |
| #68 | rape.ab,ti. | 6536 |
| #69 | mistreat*.ab,ti. | 1491 |
| #70 | molest*.ab,ti. | 1155 |
| #71 | defile*.ab,ti. | 58 |
| #72 | (adverse adj childhood adj experience*).ab,ti. | 901 |
| #73 | “ACE”.ab,ti. | 30961 |
| #74 | “violence against children”.ab,ti. | 268 |
| #75 | (abuse* adj2 spous*).ab,ti. | 356 |
| #76 | (abuse* adj2 partner*).ab,ti. | 615 |
| #77 | ((wife or wives) adj2 abuse*).ab,ti. | 153 |
| #78 | ((wife or wives) adj2 batter*).ab,ti. | 114 |
| #79 | (partner* adj2 violen*).ab,ti. | 5774 |
| #80 | (spous* adj2 violen*).ab,ti. | 145 |
| #81 | “dating violen*”.ab,ti. | 687 |
| #82 | “bully*”.ab,ti. | 3985 |
| #83 | “bullie*”.ab,ti. | 1341 |
| #84 | ((antisocial or agonis*) adj2 behavi*).ab,ti. | 4733 |
| #85 | “intimidat*”.ab,ti. | 1117 |
| #86 | “aggression*”.ab,ti. | 25105 |
| #87 | (peer* adj2 violen*).ab,ti. | 161 |
| #88 | (peer* adj2 victim*).ab,ti. | 817 |
| #89 | “juvenile delinquency” [Mesh] | 8304 |
| #90 | “bullying” [Mesh] | 4950 |
| #91 | “physical abuse” [Mesh] | 4642 |
| #92 | “domestic violence” [Mesh] | 8732 |
| #93 | “intimate partner violence” [Mesh] | 5528 |
| #94 | “spouse abuse” [Mesh] | 7203 |
| #95 | “battered women” [Mesh] | 2840 |
| #96 | “rape" [Mesh] | 9944 |
| #97 | “child abuse” [Mesh] | 30909 |
| #98 | **or/40-97** | 140321 |
|  |  |  |
| **Natural disasters string** |  |  |
| #99 | (Natural adj2 disaster*).ti,ab. | 3133 |
| #100 | (humanitarian adj2 (cris* OR emergenc* OR disaster*).ti,ab. | 603 |
| #101 | (complex adj2 emergenc*).ti,ab. | 542 |
| #102 | (displace$ adj2 (force$ or population or human or internal$)).ti,ab. | 2962 |
| #103 | avalanche*.ti,ab. | 2443 |
| #104 | earthquake*.ti,ab. | 6902 |
| #105 | temblor*.ti,ab. | 17 |
| #106 | volcano*.ti,ab. | 1896 |
| #107 | "volcanic eruption*".ti,ab. | 477 |
| #108 | supervolcano*.ti,ab. | 10 |
| #109 | flood*.ti,ab. | 13106 |
| #110 | landslide*.ti,ab. | 445 |
| #111 | mudslide*.ti,ab. | 35 |
| #112 | sinkhole*.ti,ab. | 103 |
| #113 | sink-hole*.ti,ab. | 11 |
| #114 | "limnic eruption*".ti,ab. | 4 |
| #115 | "tidal wave*".ti,ab. | 125 |
| #116 | tsunami*.ti,ab. | 2075 |
| #117 | cyclone*.ti,ab. | 1244 |
| #118 | "cyclonic storm*".ti,ab. | 12 |
| #119 | typhoon*.ti,ab. | 482 |
| #120 | hurricane*.ti,ab. | 2898 |
| #121 | "tropical storm*".ti,ab. | 147 |
| #122 | tornado*.ti,ab. | 591 |
| #123 | storm*.ti,ab. | 10979 |
| #124 | thunderstorm*.ti,ab. | 320 |
| #125 | rainstorm*.ti,ab. | 162 |
| #126 | hailstorm*.ti,ab. | 24 |
| #127 | "winter storm*".ti,ab. | 80 |
| #128 | snowstorm*.ti,ab. | 121 |
| #129 | blizzard*.ti,ab. | 190 |
| #130 | wildfire*.ti,ab. | 1328 |
| #131 | bushfire*.ti,ab. | 217 |
| #132 | "heat wave*".ti,ab. | 1128 |
| #133 | heatwave*.ti,ab. | 309 |
| #134 | drought*.ti,ab. | 15516 |
| #135 | famine*.ti,ab. | 2061 |
| #136 | "disasters" [MeSH] | 22490 |
| #137 | **or/99-136** | 76963 |
| #138 | 39 and 98 and 137 | 283 |
| #139 | limit 138 to (english language and human) | 235 |
| #140 | 139 not ((case report or editorial).ti,ab. or editorial.ptyp. or letter.ptyp. or newspaper article.ptyp.) | 235 |
