# Supplementary material for: Violence against children and natural disasters: A systematic review and meta-analysis of quantitative evidence
Source: PLoS One. 2019 May 30;14(5):e0217719. doi: 10.1371/journal.pone.0217719 (PMC6542532; doi:10.1371/journal.pone.0217719)
Supplement: S4 Table — (DOCX) [file pone.0217719.s004.docx]

**S4 Table. Descriptive analysis of included studies**

| Data source | *N* | Disaster type | Measure exposure | Measure violence |  | Perpetrator types | Respondent types | Factors used in adjustment |
| --- | --- | --- | --- | --- | --- | --- | --- | --- |
| Becker-Blease [44], United States | 1008 caregivers and 1016 10-17 year olds | Any form of disaster | Lifetime exposure to “very bad” disasters, including man-made disasters | Lifetime frequency of sexual; maltreatment; witnessing domestic violence/intimate partner violence; other major violence; any victimization events |  | Not specified | Parent/caregiver of children 2-9 years old in randomly selected households; a random selection of parent/caregiver or child between 10-17 years old | Age of child; race; number of children in household; stratified by age group |
| Biswas [52], Bangladesh | 638 mothers | Flood | Living in the most flood-affected areas from late July-early Sept 2007 | Past week binary of combined physical and emotional – sampled mid-Sept - Oct |  | Fathers; mothers | Randomly selected married women of reproductive age with at least 1 child in household | Age of respondent; religion; housing material; education; occupation; income; husband’s occupation; husband’s income; micro-credit loan; economic survival method during flood; wife abused during flood |
| Catani [50], Sri Lanka | 296 9-15 year olds | Tsunami | 5-question tsunami severity checklist – data collection date not reported | Lifetime frequency of family violence events – combined physical, emotional, sexual, and witnessing domestic violence/intimate partner violence |  | Families; household members | Randomly selected Tamil schoolchild | Age of child; gender; economic status; father alive; mother alive; father drinks alcohol; previous exposure to war |
| Curtis [45], United States | 3 months:  185 before and 259 after Hurricane Hugo; 9895 before and 10803 after Loma Prieta Earthquake; 610 before and 515 after Hurricane Andrew  6 months: 485 before and 599 after Hurricane Hugo; 20703 before and 22565 after Loma Prieta Earthquake; 1177 before and 959 after Hurricane Andrew  11 months: 1023 before and 1184 after Hurricane Hugo; 39419 before and 40537 after Loma Prieta Earthquake; 2141 before and 1788 after Hurricane Andrew | Earthquake; hurricane | Catastrophic damage to the entire parish or county and presidential disaster declaration for the county | Reported and confirmed physical; emotional; sexual; combined physical and sexual; combined physical, emotional, and sexual cases 3 months, 6 months, and 11 months pre/post disaster |  | Not specified | Administrative review of child protective service cases | Seasonal variation; national annual increases |
| Keenan [46], United States | 245 cases | Hurricane; flood | Severely affected counties with any drowning-related deaths; $\geq$ $500,000 of state/federal funds allocated for infrastructure rebuilding; and/or $\geq$ 100 home buyouts | Inflicted traumatic brain injury ~ 12 months before the disaster and the first 6 months and subsequent 6-21.5 months after the disaster |  | Not specified | Retrospective and prospective chart review of fatal or severe brain injury admissions to pediatric intensive care units and retrospective fatality chart review from the Office of the Chief Medical Examiner | Age of child; Race/ethnicity |
| Kelley [43], United States | 279 cases and 96 controls | Hurricane | Hurricane Related Traumatic Experiences (HURTE) scale – direct exposure defined by binary responses to objective or verifiable life-threatening events or 1 perceived threat question | No specified timeframe for frequencies of combined corporal punishment and yelling –sampled 3-7 months after disaster |  | Mothers | Purposeful selection of mothers of schoolchildren | None |
| Madkour [47], United States | 1637 12-18 year olds in 2005; 2018 12-18 year-olds in 2007 | Hurricane | All people in region in Aug 2005 | Last 12 months binary of physical dating violence; lifetime binary of sexual violence – sampled Aug 2004-Aug 2005 and Spring 2006-Spring 2007 |  | Boyfriend; girlfriend; not specified | Randomly selected public high school students | Age of child; race/ethnicity; gender |
| Sloand [53], Haiti | 78 12-17 year-old girls | Earthquake | None provided | No specified timeframe and question structure for physical; sexual; combined physical and sexual; combined physical, emotional, and sexual – sampled 12-36 months after disaster |  | Boyfriend; ex-boyfriend; other partners; family members; non-family members; authority figures; other person | Purposeful selection of internally displaced adolescent girls who came to local hospitals or clinics | None – sample of girls only |
| Sriskandarajah [51], Sri Lanka | 359 7-11 year olds; 108 mothers; 80 fathers | Tsunami | 5-question tsunami severity checklist | Lifetime frequency of physical; emotional; sexual; combined physical, sexual, neglect, and witnessing domestic violence/intimate partner violence – sampled in 72 months after disaster |  | Fathers; mothers | Randomly selected Tamil schoolchildren and both  caregivers/parents | None |
| Temple [48], United States | 464 14-18 year-old girls; 584 14-18 year-old boys | Hurricane; flood | Not being evacuated from affected island in Sept 2008 | Last 12-month binary of physical dating violence; lifetime binary of sexual dating violence – sampled March 2009 |  | Boyfriend; girlfriend | Purposefully selected low-income public high school students | Age of child; ethnicity; stratified by gender |
| Terranova [49], United States | 96 cases and 96 controls | Hurricane | Living within 35 miles of the eye of the storm in Sept 2005 | No specified timeframe for frequency of relational bullying victimization (emotional); overt bullying victimization (physical) – sampled May and Nov 2005 for cases and Spring 2004 and Fall 2004 for controls |  | Peers | Purposefully selected fifth grade students from a rural, economically depressed region and random selection of controls | Gender |
