# Supplementary material for: Violence against children and natural disasters: A systematic review and meta-analysis of quantitative evidence
Source: PLoS One. 2019 May 30;14(5):e0217719. doi: 10.1371/journal.pone.0217719 (PMC6542532; doi:10.1371/journal.pone.0217719)
Supplement: S5 Table — (DOCX) [file pone.0217719.s005.docx]

**S5 Table. Risk of bias results for cross-sectional and cohort studies**

| Author | Clear research question | Clear study population | Participation rate of ≥ 50% | Recruited from same population, timeframe, and consistent inclusion/  exclusion | Sample size justified, power description, or variance and effect estimates | Exposure before outcome | Timeframe under a year | Varying levels of exposure measured | Clear and consistent exposure | Exposure assessed more than once over time | Clear and consistent outcome | Assessors blinded | Loss to follow up ≤ 20% | Key confounders adjusted | Quality score |
| --- | --- | --- | --- | --- | --- | --- | --- | --- | --- | --- | --- | --- | --- | --- | --- |
| Becker-Blease [44] | Y | Y | Y | Y | N | N | N | N | Y | N | Y | N | N/A | N | 6 |
| Biwas [52] | Y | Y | Not reported | Y | Y | Y | Y | N | N | N | Y | N | N/A | Y | 8 |
| Catani [50] | Y | Y | Not reported | Y | N | N | Not reported | Y | Y | N | Y | N | N/A | N | 6 |
| Curtis [45] | Y | Y | N/A | Y | N | Y | Y | N | N | N | Y | N | N/A | Y | 7 |
| Keenan [46] | Y | Y | N/A | Y | Y | Y | Y | N | Y | N | Y | Not reported | N/A | Y | 9 |
| Madkour [47] | Y | Y | Y | Y | Y | N | N | N | N | N | Y | N | N/A | Y | 7 |
| Sloand [53] | Y | Y | N | N | N | N | N | N | N | N | Y | N | N/A | N | 3 |
| Sriskandarajah [51] | Y | Y | Y | Y | N | N | N | Y | Y | N | Y | N | N/A | N | 7 |
| Temple [48] | Y | Y | Y | Not reported | N | N | Y | N | N | N | Y | N | N/A | Y | 6 |
