# Supplementary material for: Violence against children and natural disasters: A systematic review and meta-analysis of quantitative evidence
Source: PLoS One. 2019 May 30;14(5):e0217719. doi: 10.1371/journal.pone.0217719 (PMC6542532; doi:10.1371/journal.pone.0217719)
Supplement: S6 Table — (DOCX) [file pone.0217719.s006.docx]

**S6 Table. Risk of bias results for case-control studies**

| Author | Clear research question | Clear study population | Sample size justified | Same population and timeframe | Consistent inclusion/ exclusion | Cases different from controls | *(if >100 cases or controls)* random selection | Concurrent controls | Exposure before outcome | Clear and consistent exposure | Assessors blinded | Key confounders adjusted; *(if matching)* proper statistics | Quality score |
| --- | --- | --- | --- | --- | --- | --- | --- | --- | --- | --- | --- | --- | --- |
| Kelley [43] | Y | Y | N | N | Y | Y | N | Y | N | Y | N/A | N | 6 |
| Terranova [49] | Y | Y | N | N | N | Y | Y | Y | Y | Y | N/A | Y | 8 |
