# Supplementary material for: Violence against children and natural disasters: A systematic review and meta-analysis of quantitative evidence
Source: PLoS One. 2019 May 30;14(5):e0217719. doi: 10.1371/journal.pone.0217719 (PMC6542532; doi:10.1371/journal.pone.0217719)
Supplement: S8 Table — (DOCX) [file pone.0217719.s008.docx]

**S8 Table**. **Data repositories searched**

|  | Name |
| --- | --- |
| 1 | Medline |
| 2 | PsychINFO |
| 3 | Global Health |
| 4 | Social Policy and Practice |
| 5 | Scopus |
| 6 | Cumulative Index to Nursing & Allied Health Literature (CINAHL) Plus |
| 7 | Africa-Wide Information |
| 8 | Web of Science |
| 9 | International Bibliography of Social Sciences (IBSS) |
| 10 | Index Medicus for the Eastern Mediterranean Region (IMEMR) |
| 11 | Western Pacific Region Index Medicus (WPRIM) |
| 12 | Latin American and Caribbean Index Medicus (LILACS) |
| 13 | MedCarib |
| 14 | Indexing of Indian Medical Journals (IndMED) |
| 15 | China Academic Literature Database (CNKI English) |
