# Supplementary material for: Violence against children and natural disasters: A systematic review and meta-analysis of quantitative evidence
Source: PLoS One. 2019 May 30;14(5):e0217719. doi: 10.1371/journal.pone.0217719 (PMC6542532; doi:10.1371/journal.pone.0217719)
Supplement: S9 Table — (DOCX) [file pone.0217719.s009.docx]

**S9 Table**. **Inclusion and exclusion criteria**

**Inclusion criteria**

1. Natural disasters are the exposure/treatment
2. A broad range of violence, including physical, emotional, and sexual violence, bullying, maltreatment, interpersonal violence, or witnessing domestic violence or intimate partner violence, is the outcome measure of the study
3. Person experiencing violence is a child or adolescents under 18
4. Original research published in peer-reviewed journal articles
5. All types of quantitative study design

**Exclusion criteria**

1. Articles that have gang violence, female genital mutilation (FGM) or child labour/exploitation/trafficking/marriage as outcome measures
2. Articles that use neglect as an outcome measure
3. Editorials, policy reviews or general reports that do not introduce new evidence from a specific study
4. Conference abstracts or posters
5. Literature reviews
6. Articles focused on aggression reactions or mental health sequelae in potential perpetrators without mention of an explicit act of violence against a child
7. Articles that did not define a change in magnitude or association between natural disasters and violence against children
